# Ecology of *Leptospira interrogans* in Norway Rats (*Rattus norvegicus*) in an Inner-City Neighborhood of Vancouver, Canada

**DOI:** 10.1371/journal.pntd.0002270

**Published:** 2013-06-20

**Authors:** Chelsea G. Himsworth, Julie Bidulka, Kirbee L. Parsons, Alice Y. T. Feng, Patrick Tang, Claire M. Jardine, Thomas Kerr, Sunny Mak, John Robinson, David M. Patrick

**Affiliations:** 1 School of Population and Public Health, University of British Columbia, Vancouver, British Columbia, Canada; 2 Animal Health Centre, British Columbia Ministry of Agriculture, Abbotsford, British Columbia, Canada; 3 Department of Pathology, University of British Columbia, Vancouver, British Columbia, Canada; 4 British Columbia Centre for Disease Control, Vancouver, British Columbia, Canada; 5 Department of Pathobiology, University of Guelph, Guelph, Ontario, Canada; 6 British Columbia Centre for Excellence in HIV/AIDS, St. Paul's Hospital, Vancouver, British Columbia, Canada; 7 Department of Medicine, University of British Columbia, Vancouver, British Columbia, Canada; University of Tennessee, United States of America

## Abstract

**Background:**

*Leptospira interrogans* is a bacterial zoonosis with a worldwide distribution for which rats (*Rattus* spp.) are the primary reservoir in urban settings. In order to assess, monitor, and mitigate the risk to humans, it is important to understand the ecology of this pathogen in rats. The objective of this study was to characterize the ecology of *L. interrogans* in Norway rats (*Rattus norvegicus*) in an impoverished inner-city neighborhood of Vancouver, Canada.

**Methodology/Principal Findings:**

Trapping was performed in 43 city blocks, and one location within the adjacent port, over a 12 month period. Kidney samples were tested for the presence of *L. interrogans* using PCR and sequencing. A multivariable model was built to predict *L. interrogans* infection status in individual rats using season and morphometric data (e.g., weight, sex, maturity, condition, etc.) as independent variables. Spatial analysis was undertaken to identify clusters of high and low *L. interrogans* prevalence. The prevalence of *L. interrogans* varied remarkably among blocks (0–66.7%), and spatial clusters of both high and low *L. interrogans* prevalence were identified. In the final cluster-controlled model, characteristics associated with *L. interrogans*-infection in rats included weight (OR = 1.14, 95% CI = 1.07–1.20), increased internal fat (OR = 2.12, 95% CI = 1.06–4.25), and number of bite wounds (OR = 1.20, 95% CI = 0.96–1.49).

**Conclusions/Significance:**

Because *L. interrogans* prevalence varied with weight, body fat, and bite wounds, this study suggests that social structure and interactions among rats may influence transmission. The prevalence and distribution of *L. interrogans* in rats was also highly variable even over a short geographic distance. These factors should be considered in future risk management efforts.

## Author Summary

Urban Norway rats are the source of a number of zoonotic pathogens responsible for significant human illness. *Leptospira interrogans* is one of these pathogens, and although infection in rats is asymptomatic, humans infected through exposure to the bacterium in rat urine can develop fever, renal failure, and pulmonary hemorrhage. Previous studies of *L. interrogans* in urban rats have been of limited value because they have not taken into account the complex ecology of the rat populations under study. In this study, we found that the prevalence and distribution of *L. interrogans* varied greatly between blocks in an inner-city neighborhood (reflecting that rats live in tight-knit colonies with small home ranges) and was not related to rat population size. This suggests that the *L. interrogans* ‘load’ in a block does not depend on the number of resident rats, but rather on some characteristic intrinsic to that block. Additionally, increased weight, body fat, and bite wounds were found to increase the probability of *L. interrogans* infection, suggesting that the position of a rat within the colony's social hierarchy may also influence transmission. These factors should be considered before undertaking rat control programs.

## Introduction

Norway rats (*Rattus norvegicus*) are the source of a number of zoonotic pathogens responsible for significant morbidity and mortality in cities around the world [1]. Additionally, the incidence and distribution of many rat-associated zoonoses appears to be increasing, likely due to increasing urbanization and urban poverty, which combine to promote urban rat infestations, close contact between rats and people, and transmission of zoonotic pathogens [1].

*Leptospira interrogans*, is among the most wide-spread of the urban rat-borne zoonoses, and is probably associated with the greatest human health burden, causing disease in both developing and developed nations [2]–[4]. This bacterium asymptomatically colonizes the rat kidney and is shed in the urine [3], [4], and direct or indirect contact with this urine can result in human infection [3]. In people, infection can cause fever with progression to jaundice, renal failure, and pulmonary hemorrhage [3], [4].

As with other zoonotic diseases, it is important to characterize the ecology of *L. interrogans* in the animal reservoir in order to develop an in-depth understanding of disease risk in people, and to develop intervention strategies aimed at disease prevention [5]. For example, by studying the ecology of *L. interrogans* in urban Norway rats, it may be possible to identify environmental and/or population characteristics that increase or decrease the prevalence of infection in rat populations, which will, in turn, influence the probability that people living in the same geographic area will be exposed to the bacterium. This information may facilitate the development of rat control strategies aimed at zoonotic disease prevention, specifically.

Previous studies have indicated that the prevalence of *L. interrogans* in rat populations is highly variable both among cities, and among different locations within the same city [6]–[13]. However, the factors influencing this variability in prevalence are unclear. Similarly, although some studies have found that the probability that a rat will be infected with *L. interrogans* increases with age [7], [9], [12], [13], and with female sex [9], other studies have shown no association between *L. interrogans* infection and one or both of these variables [7], [8], [12], [13]. Overall, there is a paucity of epidemiologic data regarding *L. interrogans* in urban rat populations.

This knowledge gap is a result of the fact that the complex ecology and biology of rats are seldom taken into account when studying the pathogens they carry. For example, many studies seek only to characterize *L. interrogans* prevalence and not to investigate the factors influencing prevalence [6], [10], [11], [14], [15]. Frequently, the population to which the sampled rats belong is unclear or ignored altogether, making even these simple statistics of questionable value. Meanwhile those studies with a more epidemiologic focus often lack sufficient ecologic data on the rats under study to provide an in-depth analysis of *L. interrogans* dynamics in that population [16]. Finally, some key studies have used the presence of anti-*L. interrogans* antibody as a proxy for infection [9]; however, serostatus correlates poorly with infection status in rats (i.e., many infected rats do not develop antibodies against the infecting strain of *L. interrogans*) [10], [11], [17], likely because of the close evolutionary relationship between rats and *L. interrogans* [3].

The objectives of this study were: 1) to determine whether *L. interrogans* is present in Norway rats residing in an impoverished inner-city area of Vancouver, Canada; and 2) to use ecologic data on these rat populations (collected during a year-long intensive trapping study) to characterize the prevalence and distribution of *L. interrogans* and the degree to which season and population characteristics influence the ecology of this bacterium.

Within the city of Vancouver, the impoverished Downtown Eastside was chosen as the study area because previous research had indicated that residents of poor, inner-city urban neighborhoods are at highest risk of urban, rat-associated leptospirosis [1]. This is because factors associated with poverty (i.e., infrastructural disrepair, poor hygiene, decreased health status, concurrent diseases, etc.) may result in increased rat-human contact and/or disease transmission [1]. Although there are no known cases of autochthonous leptospirosis in this neighborhood, high rates of homelessness, HIV/AIDS, and injection drug use make fever of unknown origin a common problem. This, in combination with lack of awareness regarding urban leptospirosis among health care professionals, could lead to misdiagnosis and underdiagnosis of the disease, as has been the case in other areas of the world [1].

## Methods

### Ethics Statement

This study was approved by the University of British Columbia's Animal Care Committee (A11-0087) and adhered to national guidelines set out by the Canadian Council on Animal Care (www.ccac.ca), including those pertaining to animal user training, euthanasia, protocol review, and wildlife (http://www.ccac.ca/en_/standards/guidelines).

### Sample Collection

The study area was comprised of 43 city blocks encompassing the core of Vancouver's Downtown Eastside (DTES) (N49°17′/W123°6′). Also included was an area within the port terminal, which is a center for international shipping that forms the northern border of the DTES.

In order to adequately sample each block and capture seasonal variation in rat population and disease ecology, while avoiding anthropogenic changes caused by prolonged trapping (e.g., increased population turnover, disrupted population structure, rat immigration/emigration etc.), each block (and the port site) was randomly assigned to a three-week study period over the course of one year (September 2011–August 2012). Roughly equal number numbers of blocks were studied in each season, and this random assignment suggests that there should be no systematic bias regarding which blocks were trapped in which season.

Within each block, approximately 20 Tomahawk Rigid Traps for rats (Tomohawk Live Trap, Hazlelhurst, USA) were set out along each side of the back alley that bisected the block. Traps were evenly spaced where possible, but had to be placed in a location where they did not obstruct traffic and could be secured to outdoor public property to prevent theft. At the port, traps were placed in areas where port staff had observed rats. Traps were pre-baited (filled with bait but fixed open) for one week to acclimatize rats to trapping equipment and bait, followed by two weeks of active trapping. Baits used included peanut butter, bacon fat, and oats.

Trapped rats were anesthetized with isoflurane in a rodent Inhalation Narcosis Chamber (Harvard Apparatus, Holliston, USA) prior to pentobarbital euthanasia via intracardiac injection

Morphometric data collected in the field included species (based on external morphology), sex, weight, nose-to-rump and total length, sexual maturity (females with an open vaginal orifice and males with scrotal testes were considered sexually mature), presence and number of bite wounds in the skin, and body condition on external palpation (based on the method described by Hickman et al. [18]). The date and location (block and trap) of each rat trapped was also recorded. Rats were subsequently frozen at −30°C and sent to the Animal Health Centre (AHC), British Columbia Ministry of Agriculture, Abbotsford, British Columbia for further analysis.

At the AHC, rats were thawed at 4°C and underwent a full necropsy. Necropsies were conducted from May–August 2012. During necropsy, ½ of one kidney was collected aseptically and stored at −80°C until DNA extraction (see below). Additionally, sex and sexual maturity were confirmed, pregnancy and parity in females was assessed (females that were pregnant, had visible placental scars, and/or well developed mammary tissues were considered parous), and each rat received a score based on the volume of internal fat stores (poor condition (score of 0) = minimal to no visible internal fat; moderate condition (score of 1) = moderate internal fat; good condition (score of 2) = abundant internal fat).

### *Leptospira interrogans* PCR

A total of 701 rats were trapped. Of these, 630 were tested for *L. interrogans* infection by PCR. This number was arrived at by calculating the number of rats that would need to be tested within each block and the port site in order to accurately calculate the prevalence of *L. interrogans* within that location. This calculation was performed using the sample size for proportions function in the program Ecological Methodology (Exeter Software, Setauket, USA) with an expected proportion of 50.0%, a desired margin of error of 5%, and a fixed population correction for the size of the trapped population of the block in question. For each block, the rats to be tested were selected randomly.

DNA was extracted from diluted (1∶5) and homogenized rat kidney tissues using the QiaAMP DNA Mini kit (Qiagen Inc., Canada). DNA extracts were then amplified using a real-time PCR assay which targets a 242 bp fragment of the *lipL32* gene of pathogenic *Leptospira* species [19]. The gene encodes an outer membrane lipoprotein virulence factor. Real-time PCR (RT-PCR) was performed using the Agpath-ID One-Step RT-PCR Kit (Life Technologies, Canada). Each 25 µl reaction contained 12.5 µl of 2× RT-PCR buffer, 1 µl of 25× RT-PCR enzyme, 1 µl each of forward primer (5′- AAG CAT TAC CGC TTG TGG TG -3′), reverse primer (5′- GAA CTC CCA TTT CAG CGA TT -3′) and probe (5′- FAM/AA AGC CAG GAC AAG CGC CG/BHQ1-3′), 3.5 µl nuclease-free water, and 5 µl of DNA template. Samples were run on an ABI7500 Fast PCR System. The reaction was incubated at 50°C for 2 minutes, 95°C for 10 minutes, and then amplified for 45 cycles at 95°C for 15 seconds, 60°C for 1 minute. Results were analyzed using the SDS software version 1.4. To validate this assay at our laboratory, a clone of the aforementioned *lipL32* fragment was ordered and diluted down to 3 copies per 5 µl. It was determined the RT-PCR could detect the clone at this concentration in three consecutive trials.

All primers and the probe were made by Integrated DNA Technologies (San Diego, USA) and were diluted to an initial working concentration of 20 µM and 5 µM respectively. *Leptospira interrogans* serovar Copenhageni was used as the positive control.

A subsample of 21 randomly selected RT-PCR-positive kidney samples (approximately 1/3 of all RT-PCR-positive samples) underwent conventional PCR with DNA sequencing to confirm the presence of *L. interrogans*. The conventional PCR assaytargeted a 423 bp fragment of the *lipL32* gene [20]. PCR was performed using a 25 µl reaction containing an Illustra PuReTaq Ready-to-Go PCR bead (GE Healthcare, Canada), 1 µl each of forward primer (5′- CGC TGA AAT GGG AGT TCG TAT GAT T -3′) and reverse primer (5′- CCA ACA GAT GCA ACG AAA GAT CCT TT -3′), 21 µl of nuclease-free water, and 2 µl of DNA template. Samples were run with a 5 minute initial denaturation at 95°C, followed by 50 cycles of 95°C for 1 minute, 55°C for 1 minute, 72°C for 1 minute and a final incubation of 72°C for 7 minutes to produce a 423-base pair product. All PCR samples were over-laid with two drops of mineral oil and run in a Tetrad 2 thermal cycler. PCR products were purified using Amicon Ultra 4 centrifugal filters with a 30 kDa cut-off (Fisher Scientific, Canada), diluted 1∶10 with nuclease-free water, and sequenced using the Big Dye Terminator version 3.1 Cycle Sequencing kit (Life Technologies, Canada). Each reaction contained 1 µl of 5× Big Dye Terminator Sequencing buffer, 14.4 µl of nuclease-free water, 2 µl of Big Dye Terminator, 1.6 µl of primer (diluted 1∶10). One microliter of diluted template was added for a final volume of 20 µl per reaction. Each reaction was set up in duplicate to account for forward and reverse directions. Samples were run in a Tetrad 2 thermal cycler using the following program; one cycle of 96°C for 2 minutes, then 25 cycles of 95°C for 30 seconds, 50°C for 5 seconds, and 60°C for 4 minutes. After cycling, the reactions were treated with BigDye XTerminator Purification kit (Life Technologies, Canada) as per the manufacturer's protocol. Purified sequencing products were run on an ABI 3130 Genetic Analyzer and results were interpreted using DNASTAR Lasergene 10 SeqMan Pro program. All 21 samples were identified as *Leptospira interrogans*.

### Statistical Analysis

The primary outcome variable was *L. interrogans* infection status (positive vs. negative). Given that *L. interrogans* is the species of Leptospira spp. carried by rats, and given that all sequenced PCR products were identified as *L. interrogans* (vs. other *Leptospira* spp.), a rat was considered to be infected with *L interrogans* if it was positive on the RT-PCR.

Explanatory variables that were considered included season (September–November = fall; December–February = winter; March–May = spring; June–August = summer), weight, length (nose-to-rump and total body length including tail), sex, sexual maturity (immature vs. mature), body condition as assessed by palpation in the field (score of 0–5), body condition as assessed by volume of internal fat stores on post-mortem examination (score of 0–3), presence of cutaneous bite wounds, number of cutaneous bite wounds, parity in females (nulliparous vs. parous), and pregnancy in females (see Table 1).

**Table 1:** Baseline characteristics and associations with *L. interrogans* PCR status among Norway rats.

			*L. interrogans* PCR status		
Category	Subcategory	Total (%)^a^	Positive (%)^a^	Negative (%)^a^	p – value^c^
		(n = 592)	(n = 66)	(n = 526)	
**Season**	Fall	191 (32.3)	44 (66.7)	147 (27.9)	<0.001
	Winter	113 (19.1)	14 (21.2)	99 (18.8)	
	Spring	209 (35.3)	8 (12.1)	201 (38.2)	
	Summer	79 (13.3)	0 (0)	79 (15.0)	
**Sex**	Male	325 (54.9)	39 (59.1)	286 (54.4)	0.64
	Female	259 (43.8)	27 (40.9)	232 (44.1)	
**Sexual maturity**	Mature	328 (55.4)	64 (97.0)	264 (50.2)	<0.001
	Immature	205 (34.6)	1 (1.5)	204 (38.8)	
**Weight** (g)	Median (IQR)	123.7 (63.6–252.8)	302.5 (252.0–344.9)	96.2 (59.2–221.2)	<0.001
**Nose-to-rump length** (cm)	Median (IQR)	16.5 (13.0–21.0)	21.8 (21.0–23.0)	15.5 (13.0–20.0)	<0.001
**Total length** (cm)	Median (IQR)	31.5 (25.2–39.0)	40.8 (39.0–42.7)	29.2 (25.0–37.9)	<0.001
**Body condition score**	Median (IQR)	2.5 (2.0–3.0)	2.5 (2.0–3.0)	2.5 (2.0–3.0)	0.81
**Fat score** (categorical)	Poor	251 (42.4)	2 (3.0)	249 (47.3)	<0.001
	Moderate	169 (28.5)	18 (27.3)	151 (28.7)	
	Good	156 (26.4)	46 (69.7)	110 (20.9)	
**Fat score** (continuous)	Median (IQR)	1.0 (0.0–2.0)	2.0 (1.0–1.67)	1.0 (0.0–1.0)	<0.001
**Wound presence**	No	477 (75.5)	29 (43.9)	418 (79.5)	<0.001
	Yes	145 (24.5)	37 (56.1)	108 (20.5)	
**Wound number**	Median (IQR)	0.0 (0.0–0.0)	1.0 (0.0–2.0)	0.0 (0.0–0.0)	<0.001
**Parous** b	No	180 (69.5)	6 (22.2)	174 (75.0)	<0.001
	Yes	73 (28.2)	20 (74.1)	53 (22.8)	
**Pregnant** b	No	227 (87.6)	18 (66.7)	209 (90.1)	0.001
	Yes	26 (10.0)	8 (29.6)	18 (7.8)	

a Frequencies and percentages may not add to 100% because of exclusion of rats with missing data for the variable in question.

b Females only (n = 259).

c Determined using the Chi-squared test, Fisher's exact test, or Welch's t-test, where appropriate.

To identify characteristics associated with *L. interrogans* infection status, the distribution of the explanatory variables were examined among the sample as whole, as well as separately for *L.interrogans*-positive and -negative rats. Simple logistic regression was used to examine relationships between *L*. *interrogans* infection and each of the explanatory variables. Variables that were significantly associated with *L. interrogans* infection at an alpha level of ≤0.10 were considered for inclusion in a multiple logistic regression (MLR) model. Spearman's rank correlation was used to confirm that none of the variables included in the final model were strongly collinear (rho >0.8). For collinear variables (e.g., weight and length), models were created using each of the variables independently and compared. The final MLR model was selected using Aikake's Information Criterion (AIC) to balance model fit and parsimony. Subsequently, the variables included in the final logistic regression model were entered into a generalized lineal mixed (GLM) model to control for the effect of block, and AIC was used to select the final GLM model. By creating both MLR and GLM models, we were able to appreciate the effect of cluster-control.

The dataset was then stratified and the final MLR and GLM models run in males vs. females to identify effect modification by sex. The female dataset was also used to examine the effect of parity and pregnancy in bivariable and multivariable models.

All statistical analyses were conducted using R (R Development Core Team, Vienna, Austria). For multivariable models, individuals with missing data for one or more of the variables under study were excluded.

### Spatial Analysis

The location of each trap within the 43 block area of the DTES, and the number of rats caught in each trap that were *L. interrogans*-positive and -negative were mapped using ArcGIS 10.0 (ESRI, Redlands, USA). This information was imported into SaTScan (Boston, USA) for cluster analysis using a purely spatial Bernoulli model and scanning for areas with high and low rates of *L. interrogans* infection using a circular window with a maximum spatial cluster size of 50% of the population at risk. Clusters identified by SaTScan were visualized in ArcGIS. The port site was excluded from this analysis because trapping took place at multiple levels within a single geographic foot-print (which is difficult to represent in a two dimensional map) and because trapping was somewhat more opportunistic (vs. systematic) compared to the blocks.

## Results

Among the 630 rats tested for *L. interrogans* by PCR, only 38 (6.0%) were black rats, while the remainder were Norway rats. None of these 38 black rats were positive for *L. interrogans*, therefore black rats were removed from the analytic sample. The following statistics pertain only to Norway rats (n = 592), henceforth referred to as rats.

The majority of rats were trapped in the spring, followed by the fall, winter, and summer (see Table 1). There were slightly more males (54.9%) than females and over half of the rats were sexually mature (55.4%). The average (median) body weight was 123.7 g, and the average nose-to-rump and total length (including the tail) was 16.5 cm and 31.5 cm, respectively. The average body condition score as judged by palpation in the field was 2.5 out of 5 and 42.4% of rats were judged to have poor internal fat stores, while 28.5% and 26.4% had moderate and good fat stores, respectively. The average number of bite wounds per rat was 0.0 as the majority of rats (75.5%) had no wounds.

**Figure 1:**
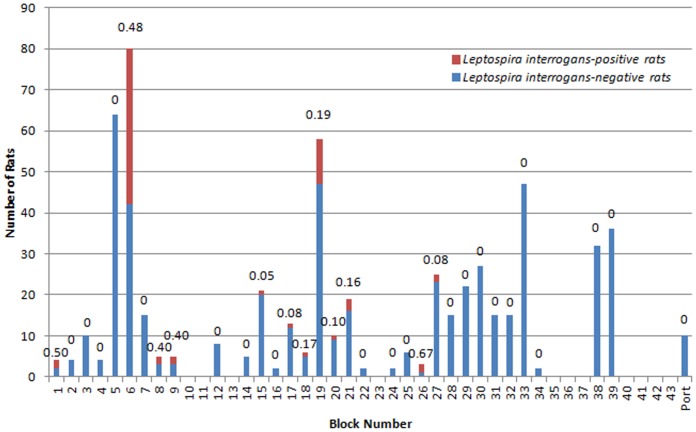
Number of *L. interrogans*-positive vs. -negative Norway rats in each study location. Prevalence of *L. interrogans* is noted above each bar.

The overall prevalence of *L. interrogans* was 11.1% (66/592). However, there was marked variation in the prevalence of *L. interrogans* by block, with prevalence ranging from 0% to 66.7% (see Figure 1).

On bivariable analysis, the odds of being *L. interrogans*-positive was less in rats caught in the spring (OR = 0.14, 95% CI = 0.06–0.28) or winter (OR = 0.47, 95% CI = 0.24–0.89) compared to the fall (see Table 2). The odds of being *L. interrogans* positive was also less in sexually immature rats compared to mature rats (OR = 0.02, 95% CI = 0.001–0.09). The odds of being *L. interrogans*-positive increased with increasing weight (OR = 1.15, 95% CI = 1.12–1.19), nose-to-rump length (OR = 1.65, 95% CI = 1.46–1.90), and total length (OR = 1.32, 95% CI = 1.23–1.43). Rats with bite wounds had greater odds of being *L. interrogans*-positive compared to rats with no bite wounds (OR = 4.94, 95% CI = 2.91–8.44), and the odds of being *L. interrogans*-positive increased with increasing number of bite wounds (OR = 1.45, 95% CI = 1.25–1.69). Body condition score was not significantly associated with *L. interrogans* infection status (OR = 1.04, 95% CI = 0.73–1.49), however, the odds of being *L. interrogans*-positive was greater in rats with a greater volume of internal fat (OR = 5.12, 95% CI = 3.40–8.13; note that fat score was entered into the multivariable models as a continuous variable for better model fit).

**Table 2:** Unadjusted and adjusted odds ratios for being *L. interrogans* PCR-positive among Norway rats.

		Unadjusted		Adjusted (MLR^e^)		Adjusted (GLMM^e^)	
Category	Subcategory	OR^a^	95% CI	OR	95% CI	OR	95% CI
**Season**	Fall	Ref^b^		Ref		–	–
	Spring	0.14	0.06–0.28	0.19	0.07–0.45	–	–
	Summer	NA^c^	NA	NA	NA	–	–
	Winter	0.47	0.24–0.89	0.31	0.14–0.67	–	–
**Sex**	Male	Ref		–	–	–	–
	Female	0.85	0.50–1.43	–	–	–	–
**Maturity**	Mature	Ref				–	–
	Immature	0.02	0.001–0.09			–	–
**Wound presence**	No	Ref		–	–	–	–
	Yes	4.94	2.91–8.44	–	–	–	–
**Wound number**		1.45	1.25–1.69	1.19	0.99–1.41	1.20	0.96–1.49
**Weight** (10 g)		1.15	1.12–1.19	1.12	1.07–1.17	1.14	1.07–1.20
**Nose-to-rump length** (cm)		1.65	1.46–1.90	–	–	–	–
**Total length** (cm)		1.32	1.23–1.43	–	–	–	–
**Body condition score**		1.04	0.73–1.49	–	–	–	–
**Fat score** (categorical)	Poor	Ref					
	Moderate	14.8	4.21–94.20	–	–	–	–
	Good	52.1	15.72–322.37	–	–	–	–
**Fat score** (continuous)		5.12	3.40–8.13	2.10	1.20–3.78	2.12	1.06–4.25
**Parous** d	No	Ref		–	–	–	–
	Yes	10.94	4.41–31.22	–	–	–	–
**Pregnant** d	No	Ref		–	–	–	–
	Yes	5.16	1.90–13.30	–	–	–	–

a Odds ratio (OR) with 95% confidence interval (CI).

b Reference category.

c Insufficient power to provide a meaningful estimate for this category.

d Females only (n = 259).

e MLR = Multiple logistic regression model, GLMM = Generalized linear mixed model.

In the final multivariable logistic regression model, season (OR = 0.19, 95% CI = 0.07–0.45 for spring vs. fall and OR = 0.31, 95% CI = 0.14–0.67 for winter vs. fall), wound number (OR = 1.19, 95% CI = 0.99–1.41), weight (OR = 1.12, 95% CI = 1.07–1.17), and fat score (OR = 2.10, 95% CI = 1.20–3.78) were retained (see Table 2). The relationship between these variables and *L. interrogans* positivity was in the same direction but of decreased magnitude compared to bivariable analysis. After controlling for clustering by block, only weight, wound number, and fat score were retained. In the final GLM model, the odds of being *L. interrogans*.-positive increased with increasing weight (OR = 1.14, 95% CI = 1.07–1.20), volume of internal fat (OR = 2.12, 95% CI = 1.06–4.25), and number of bite wounds (OR = 1.20, 95% CI = 0.96–1.49), although the last relationship was only marginally significant.

Upon stratifying the models by sex, there was no apparent difference in the relationship between the explanatory variables and *L. interrogans* positivity in males vs. females. Among females, the odds of being *L. interrogans*-positive was higher in rats that were parous (vs. non- parous) and pregnant (vs. non-pregnant). However, neither pregnancy nor parity was significantly associated with *L. interrogans* positivity, or improved model fit, once incorporated into a multivariable model.

**Figure 2:**
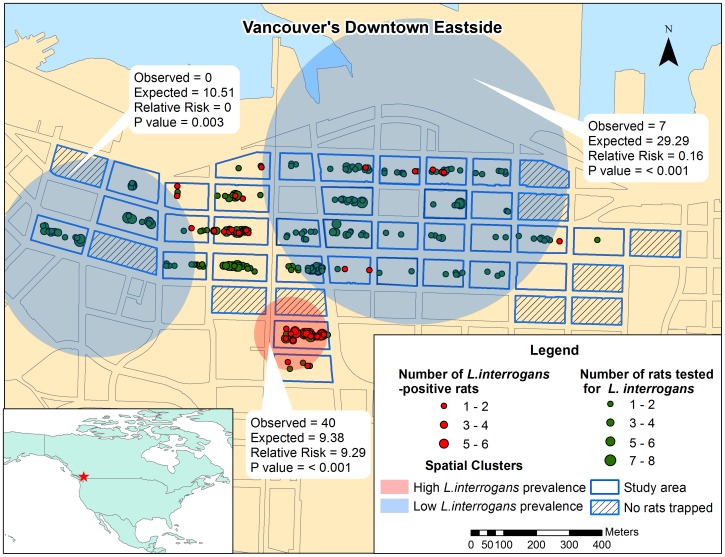
Distribution of *L. interrogans*-positive Norway rats and clusters of high and low *L. interrogans* prevalence. Observed vs. expected number of *L. interrogans*-positive Norway rats with relative risk and p values noted for each cluster.

In the final GLM model, the estimated variance for the random effect of block was 4.34, indicating that block of origin had a significant impact on *L. interrogans* infection status. Geographic clustering of cases was also evident on spatial analysis, which identified one cluster (very spatially compact) with greater than expected prevalence of *L. interrogans* infection and two clusters with lower than expected prevalence of *L. interrogans* spp. infection (see Figure 2).

## Discussion

Overall, this study demonstrates that *L. interrogans* is present in Norway rats within this inner-city neighborhood; however, the distribution of *L. interrogans* is not uniform. Previous studies have shown that the prevalence of *L. interrogans* in rats may vary among different locations within a city [12]. However, this study showed that marked clustering can take place even over a very small geographic distance within a single neighborhood.

The presence of clustering is consistent with what is known about the ecology of rats in urban centers. The size of a rat's home range is determined by the availability of suitable harborage and food sources, social pressure from conspecifics or other rat species, and the presence of barriers to rat movement [21], [22]. In urban centers, the ubiquity of resources and the barrier-effect of roadways combine to result in small home ranges that are often limited to a city block [22], [23]. In the absence of drastic changes to the environment, long distance migration is uncommon [22]. Additionally, rats are both colonial and territorial [24], [25]; therefore, conspecifics residing in the same geographic area are likely to be members of the same colony and interact. For these reasons it is not surprising that the presence of numerous functionally distinct rat colonies would lead to heterogeneity in pathogen distribution within an urban ecosystem.

Clustering, however, has significant implications for analysis and interpretation of epidemiologic data in rats. It suggests that aggregated city-level pathogen prevalence, as is often reported [7], [9], [11], is not the best measure of *L. interrogans* frequency. Rather, it may be more valid to measure prevalence at the level of the colony or block. Clustering must also be taken into account when attempting to identify factors that influence the prevalence *L. interrogans* in order to avoid bias associated with variation in distribution of disease determinants among clusters. For example, in this study, season appeared to be a predictor of *L. interrogans* status in the MLR model. After controlling for clustering, however, the effect of season was no longer significant. This suggests that the apparent effect of season may have been an artefact resulting from the fact that blocks with a high *L. interrogans* prevalence were trapped in one particular season.

In the final GLM model, body weight was positively associated with *L. interrogans* infection status. Specifically, a 10 g increase in the weight of a rat was associated with a 14% increase in the odds of that rat of being *L. interrogans*-positive. This association has been noted in previous studies of *L. interrogans* in urban rats [9], [12], [13], and was presumed to be a result of the fact that the older the animal (as weight is a good proxy for age [22]), the greater the likelihood that it will become exposed to and infected with *L. interrogans* [12], [13]. However, given that *L. interrogans* is thought to be transmitted through urine [3], and given the colonial nature of rats and the opportunity for direct and indirect exposure to conspecifics from birth [25], it is surprising that young rats do not become exposed earlier in life. In this study, the median weight of *L. interrogans*-positive rats was 302.5 g, which is consistent with the weight of an adult rat, compared to 96.2 g for *L. interrogans*-negative rats, which is consistent with the weight of a juvenile [22]. This suggests that the mechanism of *L. interrogans* infection in rats may be more complex than random environmental exposure, or else one would expect to see infection occur soon after young leave the nest.

This complexity is supported by the fact that the volume of internal fat, independent of weight, was also significantly associated with *L. interrogans* positivity, and number of bite wounds was marginally significant. Specifically, the odds of being *L. interrogans*-positive were more than twice as high for a rat with higher fat score compared to a rat with a lower fat score, and increased by 20% with each bite wound. This may suggest that *L. interrogans* transmission has a social or behavioral aspect. For example, dominant rats have greater access to food resources and are more likely to engage in aggressive behavior compared to non-dominant rats [22], [25], [26]. This could result in dominant rats having greater internal fat stores and a higher incidence of bite wounds. Additionally, dominant rats are generally heavier that subordinate rats [22]. It may therefore be the case that body fat, bite wounds, and/or weight are markers of social hierarchy, and the relationship between these variables and *L. interrogans* infection status is, at least in part, a result of behavior. For example, dominant rats may exhibit more exploratory behavior and have more contact with conspecifics compared to subordinates [25], both of which could increase the opportunity for exposure to *L. interrogans*.

Some studies, however, have found that high-ranking rats actually have fewer bite wounds compared to low-ranking rats [22], presumably because the low-ranking rats are more frequently on the ‘losing end’ of intraspecific conflicts. If this was the case then the association between number of bite wounds and *L. interrogans* positivity observed in this study might suggest that biting could be a method of *L. interrogans* transmission among rats. *Leptospira interrogans* has been transmitted from a rat to a human through biting [27], and biting is an important mode of rat-to-rat transmission of Seoul hantavirus, another rat-associated pathogen [28].

The potential role of maternal antibody transfer in mediating *L. interrogans* infection dynamics also deserves consideration. Maternal antibody has been shown to prevent persistent infection with Seoul hantavirus in rats, and may persist for up to 5 months of age [29]. Maternal transfer of antibody could also delay infection with *L. interrogans*, and might partially explain why infection was uncommon in juveniles. That being said, *L. interrogans* are extremely well adapted to their reservoir hosts [3], and many rats infected with *L. interrogans* do not have detectable circulating antibody [10], [11]. For this reason further study is needed to determine if maternal antibody plays a role in the ecology of *L. interrogans* in rats.

One limitation of this work is the fact that very few black rats were included in this study sample. This could be a result of the trapping methodology (i.e., trapping on the ground in outdoor areas), which may bias towards trapping Norway vs. black rats. This is because Norway rats more commonly reside in outdoor underground burrows, while black rats are more commonly found in the upper levels of man-made structures [30]. Indeed, previous studies have found that placing traps in elevated positions inside structures (e.g., in the roof) increased catch success for black rats [31]. Alternatively, it could be the case that Norway rats are truly more ubiquitous in this urban environment compared to black rats. Norway rats, as a species, are larger and more aggressive compared to black rats, and tend to displace black rats where the two species coexist [22], [24], [30]. In this study, none of the black rats were infected with *L. interrogans*, which may reflect either inadequate sampling or a low prevalence of disease in this species. Interestingly, other studies of *L. interrogans* in Norway and black rats have also found a comparatively low prevalence of infection in the black rats [16]. This could be a result of the tendency of black rats to nest off the ground (as opposed to the ground-burrowing Norway rat) [30], which could decrease their exposure to urine-borne pathogens. Given the fact that Norway and black rats differ in many aspects of their ecology [26], [30], it is possible that the prevalence and epidemiology of *L. interrogans* differs between these two species. Future studies should seek to study *L. interrogans* in black rats, specifically.

In this study, we were able to show that the number of *L. interrogans*-infected rats in a block was not related to the size of the resident rat population. In other words, contrary to conventional wisdom, a larger rat infestation does not necessarily equal a larger *L. interrogans*-associated disease risk. Rather, it appeared that there was some block-level characteristic(s) that had a significant impact on *L. interrogans* prevalence. Although we could not identify these characteristics, it is our suspicion that they are likely features of the block environment.

Rats themselves are strongly influenced by the environment in which they reside [31], [32]; therefore it seems likely that the environment would also influence *L. interrogans* ecology, either directly, or indirectly through its effect on population ecology. The environment within our study area, however, is relatively uniform, being composed primarily of high-density residential and commercial properties with minimal green space. There were no obvious systematic differences between blocks with a high and low prevalence of *L. interrogans*. It is likely therefore, that small differences in block composition (e.g., availability of soil for burrowing and volume of exposed garbage), rather than high-level ecosystem differences, are influencing the distribution of *L. interrogans* in this environment. Future studies of *L. interrogans* in rats should seek to quantify these subtle differences and relate them to the *L. interrogans* ecology.

The findings from this study have potential public health significance as they suggest that the risk of a person being exposed to rat-associated *L. interrogans* is highly heterogeneous across the urban environment and not necessarily dependent on the number of rats infesting a particular area. Additionally, the association between *L. interrogans* and weight seems to suggest that established populations with a high proportion of adults pose a greater risk than populations with mostly juvenile rats. Given that large, dominant rats may be preferentially removed by trapping and poisoning campaigns, due to their propensity towards exploratory behavior and competitive exclusion of subordinates [26], [33], it may be the case that trapping and poisoning may preferentially remove *L. interrogans*-infected rats. That being said, rodent control activities may also disrupt social structures, trigger long-distance rat migrations, and result in intraspecific antagonism [22], [33], any or all of which could have unpredictable effects on *L. interrogans* dynamics. Indeed in other situations, attempts to control disease in wild animals through culling have caused a paradoxical increase in disease prevalence by disrupting otherwise stable populations and thereby increasing disease transmission [34]. For this reason future studies should aim to determine the impact of rat control strategies on *L. interrogans* dynamics in urban rats.

In conclusion, this study shows that the ecology of *L. interrogans* in Norway rats is inextricably intertwined with rat population ecology and that further study is needed in order to identify micro-environmental factors influencing *L. interrogans* prevalence in rats, to determine if the ecology of this bacterium varies among different rat species, and to determine how rat control strategies might impact *L. interrogans* dynamics in cities. Given the increasing incidence of urban rat-associated leptospirosis in people [1], and given that the ecology of *L. interrogans* and its rat reservoir hosts have a significant impact on the risk of transmission to people [1], it is clear that these further studies will be necessary if we are to proactively confront this public health threat.

## Supporting Information

Dataset S1 **Raw data used for the statistical analysis in this study.**
(XLS)
